# Ophthalmologic Findings in an Induced Model of Holoprosencephaly in Zebrafish

**DOI:** 10.1002/cne.70113

**Published:** 2025-11-09

**Authors:** Johannes Bulk, Valentyn Kyrychenko, Stephan Heermann

**Affiliations:** ^1^ Department of Molecular Embryology, Institute of Anatomy and Cell Biology, Faculty of Medicine University of Freiburg Freiburg Germany; ^2^ Faculty III – Health, Medical & Life Sciences Furtwangen University Furtwangen Germany

**Keywords:** anophthalmia, BMP, *bmp4*, crypt‐oculoid, cyclopia, holoprosencephaly

## Abstract

Holoprosencephaly (HPE) is the most frequent developmental disorder of the forebrain. In this condition, the separation of the early precursor domains is hampered. A spectrum of clinical manifestations can be observed, for example, severe forms like alobar HPE and less severe forms like lobar HPE. Ophthalmologic findings that accompany HPE also occur as a spectrum that ranges from ocular hypotelorism and synophthalmia to cyclopia and anophthalmia. In this brief analysis, we made use of a recently established zebrafish model of HPE. This model is based on experimental BMP ligand induction that resulted in anophthalmia. We attenuated the induction protocol to investigate whether the ophthalmologic phenotype can also be attenuated. We found a spectrum of ocular phenotypes: ocular hypotelorism, cases of synophthalmia, and cyclopia.

## Graphical abstract

We used a zebrafish (*Danio rerio*) model to variably induce *bmp4* during embryonic development. We show ophthalmologic phenotypes of variable severity depending on the length and onset of *bmp4* inductions. We find phenotypes like ocular hypotelorism, synophthalmia, cyclopia, and anophthalmia, which are described to be part of the HPE spectrum.

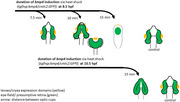

## 1 Introduction

Holoprosencephaly (HPE) is the most frequent developmental pathology of the forebrain (Malta et al. 2023; Matsunaga and Shiota 1977; Pineda‐Alvarez et al. 2011), in which the splitting of precursor domains of the anterior neural plate (ANP) is hampered. It is well established that specific genes, if mutated, as well as specific environmental factors, enhance the risk for HPE (Addissie et al. 2021; Roessler et al. 2018). Nevertheless, the exact role and interplay of these genes and factors during HPE development, and importantly, HPE development by itself, are not perfectly understood. For example, not all patients who suffer from HPE show the same specific phenotype, but rather a huge spectrum of phenotypes. This spectrum was divided into groups of severe forms such as alobar HPE, intermediate forms such as semilobar HPE, and less severe forms such as lobar HPE (Gomez et al. 2024). As both the prospective telencephalic lobes as well as both retinae derive from the ANP, it is consistent that ophthalmologic phenotypes are a part of HPE (Pineda‐Alvarez et al. 2011). Such ophthalmologic phenotypes also occur as a spectrum. This ranges from coloboma, ocular hypotelorism, and synophthalmia to cyclopia and anophthalmia (Fallet‐Bianco 2018; Pineda‐Alvarez et al. 2011). Fundamental discoveries regarding the role of BMP signaling during early forebrain and eye development have been made in the mouse and chicken models. Analyses of double knockout mice of two BMP antagonists, noggin and chordin, have been carried out. They revealed that the formation of the forebrain was affected variably, which resulted in, for example, cyclopia and aprosencephaly (Bachiller et al. 2000; Klingensmith et al. 2010). A knockout of noggin alone resulted in a milder form of HPE (Lana‐Elola et al. 2011). In chicken, BMP application via soaked beads resulted in cyclopia (Golden et al. 1999). These effects are likely mediated by somehow affecting signals emanating from the ventral region (shh) or their targets within the ANP. This is classically considered to be crucial for forebrain splitting (Chiang et al. 1996; Roessler et al. 1996) and likely affects the subduction of the presumptive hypothalamic domain (England et al. 2006). Interestingly, however, the roof plate was also observed to affect the splitting of the forebrain (Cheng et al. 2006). This was found to be linked likely to a distinct form of HPE, the middle interhemispheric variant. The roof plate is an important source for BMP ligands (Furuta et al. 1997). Consistently, a double knockout of two BMP receptors, Bmpr1a and Bmpr1b, affected dorsal aspects of the developing midline, accompanied by microphthalmia and an HPE phenotype that was suggested to be the middle interhemispheric variant, and a new class of HPE was proposed (Fernandes et al. 2007).

We used zebrafish (*Danio rerio*) as a model and recently found that BMP antagonism is crucial for various consecutive steps of eye development. The BMP antagonists *Nog2*, *chrd*, *fsta*, and *grem2b* were found to be expressed in or adjacent to the ANP, and an induction of the BMP ligand *bmp4* at 8.5 hpf was sufficient to hamper ANP splitting, which resulted in a severe form of HPE and anophthalmia (Bulk et al. 2023). In consecutive stages of development, we found *fsta* expressed in the optic vesicle and optic cup, and inductions of *bmp4* at different stages of development hampered optic fissure formation and optic‐vesicle‐to‐optic‐cup transformation, resulting in a “morphogenetic coloboma” (Eckert et al. 2019; Heermann et al. 2015). At an even later stage of development, we found *grem2b* and *fsta* expressed in the margins of the optic fissure. An induction of *bmp4* at this stage resulted in a “fusion‐inhibited coloboma” (Knickmeyer et al. 2018). Based on our findings, we propose a continuous gradient of these forebrain and eye developmental processes, all of which are crucially facilitated or protected by BMP antagonism. Disturbances of this BMP antagonism could explain well different ophthalmologic phenotypes associated with HPE, including coloboma (Pineda‐Alvarez et al. 2011).

In this brief study, we dive further into the morphogenetic and molecular background of the new phenotype previously described by us. We made use of the aforementioned model of *bmp4* induction in zebrafish, in which an induction at 8.5 hpf resulted in anophthalmia and a “crypt‐oculoid” of retinal precursors stuck in the forebrain (Bulk et al. 2023). Now, we attenuated the induction protocol to investigate whether, instead of anophthalmia, other ophthalmologic phenotypes could be observed. Following attenuated induction regimes, we found ocular hypotelorism, synophthalmia, and cyclopia. Furthermore, we evaluated the development of lens tissue in our observed phenotypes by addressing the expression of *cryaa*, a gene coding for a crystallin protein, important for lens development and function. The effect of *bmp4* on the basal constriction of the retinal progenitors during optic cup formation was examined by us through a delayed *bmp4* induction at 10.5 hpf.

## 2 Results

### 2.1 Attenuated *bmp4* Induction Results in a Spectrum of Eye Phenotypes Including Cyclopia

**FIGURE 1:**
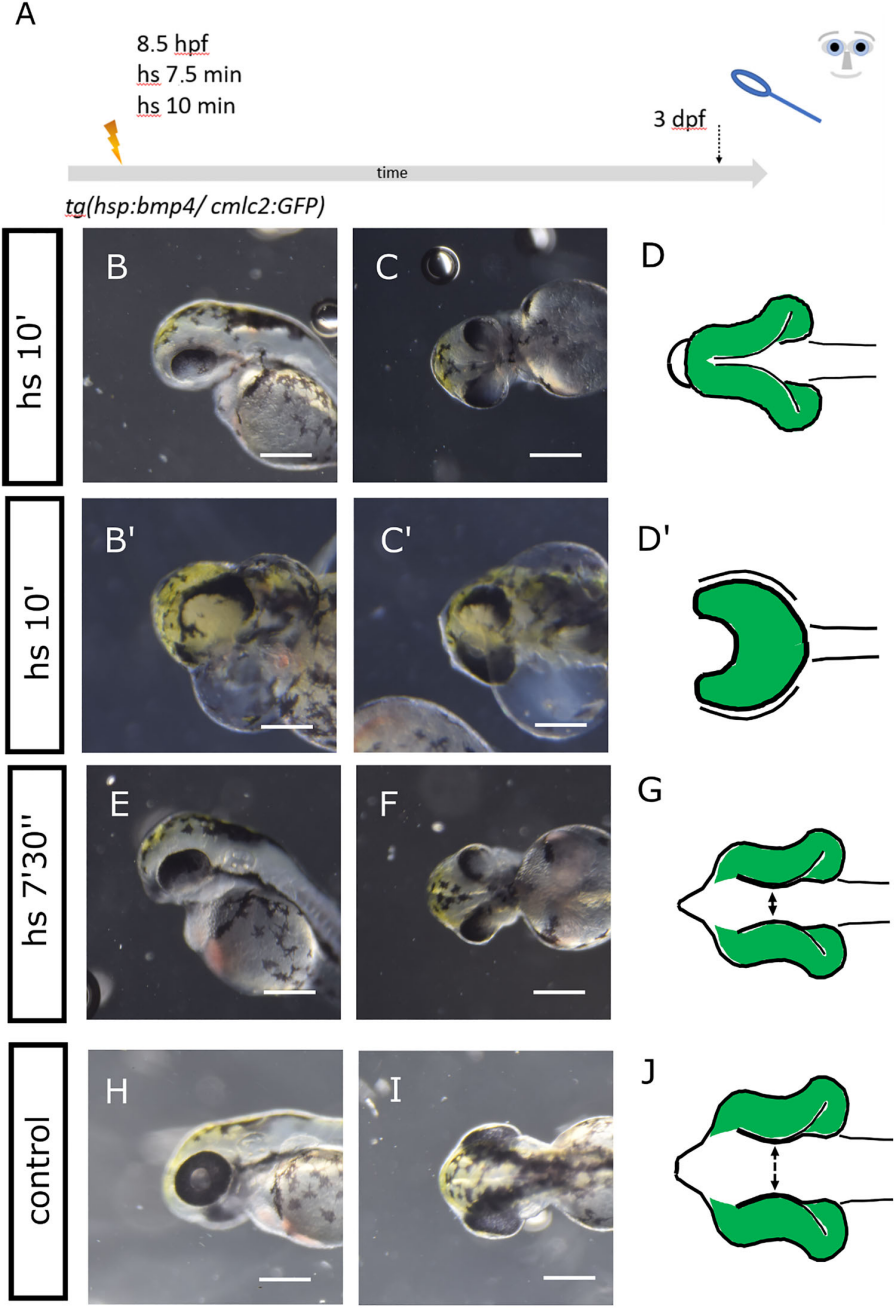
Attenuated *bmp4* induction. (A) Summary of experimental procedure: embryos were heat‐shocked at 8.5 hpf for 7.5 or 10 min and analyzed at 3 dpf. (B–Dʹ) *bmp4* induction for 10 min. Embryos show a spectrum of phenotypes. Frequently, two optic cups are seen that are fused in the middle, synophthalmia (B, C). However, cases of cyclopia with severe pericardial effusion (*pe*) can also be seen (Bʹ and Cʹ). (D and Dʹ) Schemes of phenotypes. The scheme in D matches with image C, and Dʹ matches with Cʹ. (B) Lateral view, head left. (C, Cʹ, Bʹ, D, and Dʹ) Ventral view, head left. (E–G) *bmp4* induction for 7.5 min. The embryos develop ocular hypotelorism. (G) Scheme of phenotype. (E) Lateral view, head left. (F) Ventral view, head left. (H–J) Control embryo. (J) Scheme of phenotype. (H) Lateral view, head left. (I) Dorsal view, head left. Scale bars indicate 250 µm. The dotted lines indicate the eye domain; *y*: yolk sac. The green color in the schematic drawings indicates eye tissue of neuroectodermal origin.

We used a transgenic zebrafish line that expresses *bmp4* after induction via heat shock (*Tg(hsp70l:bmp4,myl7:EGFP*)). The genetic construct also contains a reporter that drives the expression of GFP within the heart (myosin light chain 7 (myl7):GFP). This line was established and used previously (Bulk et al. 2023; Knickmeyer et al. 2018). *Bmp4* induction at 8.5 hpf for 15 min resulted in a severe form of HPE with anophthalmia (Bulk et al. 2023). Notably, a “crypt‐oculoid” of retinal precursor cells was found inside the forebrain. We now attenuated the induction regime to address whether, instead of anophthalmia, other ocular phenotypes could be observed. We performed heat shocks at 8.5 hpf that were shorter in duration (Figure 1, scheme of experimental design) compared with the protocol we used previously. We induced *bmp4* expression for 10 min (100%, 186 embryos with *myl7:GFP* expression were phenotypic; 100%, 198 embryos that did not express *myl7:GFP* were not phenotypic) and 7.5 min (100%, 40 embryos with *myl7:GFP* expression were phenotypic; 97%, 36 embryos that did not express *myl7:GFP* were not phenotypic; 3%, one embryo that did not express *myl7:GFP* showed ocular hypotelorism and coloboma). A summary of metadata can be found in the Supporting Information Table. The induction for 10 min resulted in a spectrum of phenotypes. Its range led from synophthalmia, an incomplete separation of the eyes with two yet distinguishable optic cups (Figure 1B–D, please see H–J as control; Figure S1), to cyclopia (Figure 1Bʹ–Dʹ, please see H–J as control). The cyclopic fish displayed a severe pericardial effusion as in the HPE/anophthalmia phenotype previously described. We observed a continuous distribution of phenotypes between those two parameter values. Of a total of 186 fish observed, we can account for seven synophthalmic and three entirely cyclopic fish. The induction for 7.5 min resulted in ocular hypotelorism, a reduced distance between the eyes (Figure 1E–G, please see H–J as control).

### 2.2 *Cryaa* Expression in *bmp4*‐Induced Embryos

**FIGURE 2:**
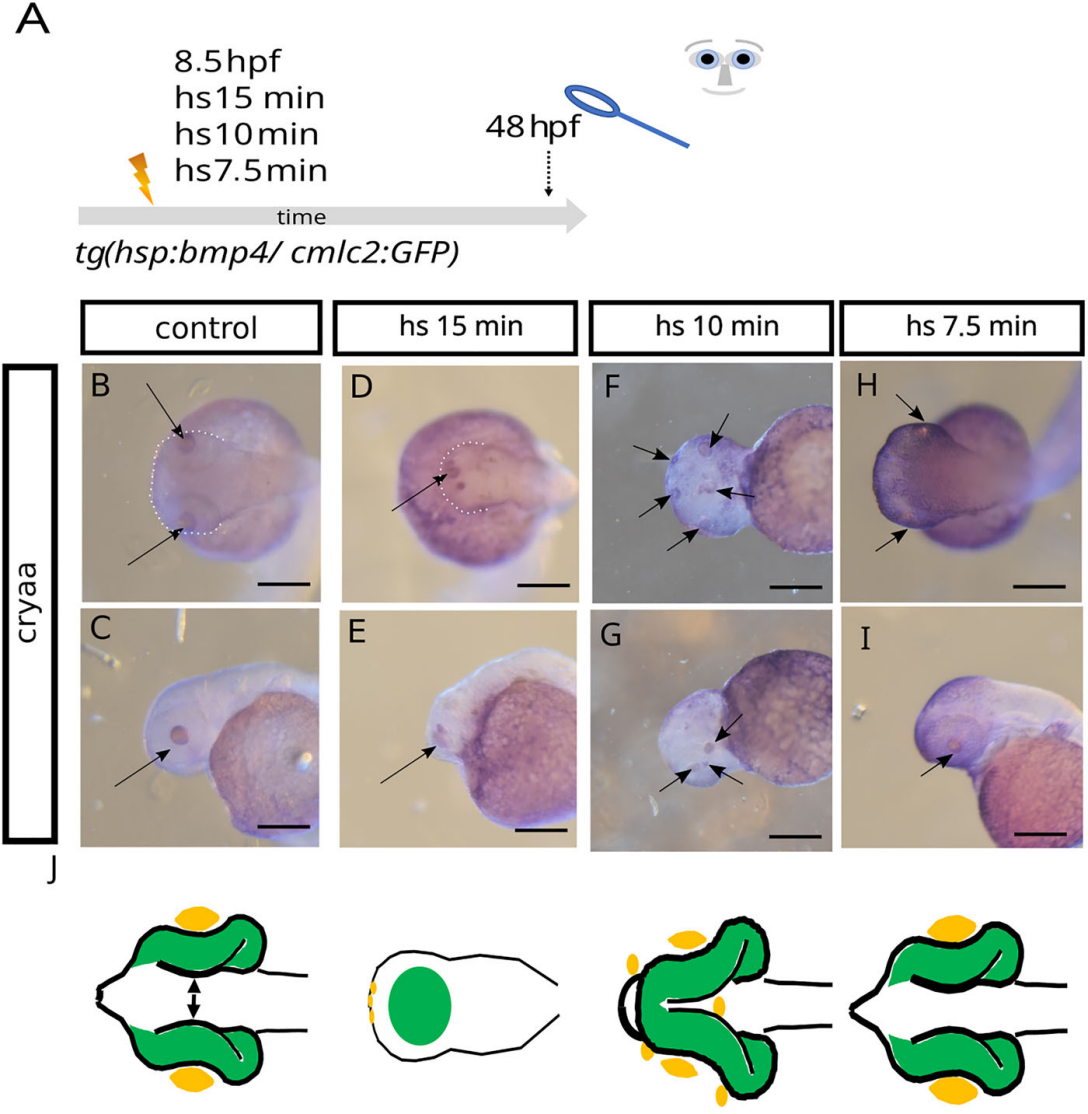
*Cryaa* expression in embryos after attenuated *bmp4* induction. (A) Summary of experimental procedure: embryos were heat‐shocked at 8.5 hpf for 15, 10, and 7.5 min and analyzed at 48 hpf. (B–E) WMISH for *cryaa* at 48 hpf. (B, C) Control embryo. (B) Dorsal view, head (dotted line) left (arrow points at lenses). (C) Lateral view, head left (arrow points at lens). (D, E) *bmp4*‐induced embryo (15 min). (D) Dorsal view, head left. (E) Lateral view, head (dotted line) left. No eyes or lenses were formed. Multiple small *cryaa*‐positive domains are visible at the tip of the head (arrows in D and E). (F, G) *bmp4*‐induced embryo (10 min). (F) Ventral view, head left. (G) Lateral view, head left (arrows point at sites of *cryaa* expression). (H, I) *bmp4*‐induced embryo (7.5 min). (H) Ventral view, head left. (I) Lateral view, head left (arrows point at sites of *cryaa* expression). Scale bars indicate 200 µm. The dotted lines indicate the outlines of the heads. *y*: yolk sac. *K*: schematic representations of phenotypes. Green: eye tissue (of neuroectodermal origin); yellow: lens tissue (of ectodermal origin).

Given the variety of observed eye phenotypes, we were interested in a more thorough examination of anatomical structures. In particular, we wanted to observe the distribution of lens tissue in our phenotypes, since the existence of one median lens provides a straightforward possibility of differentiating cyclopia from synophthalmia. An interaction between pre‐lens ectoderm and optic vesicle is furthermore fundamental for the transformation of an optic vesicle into an optic cup (Hyer et al. 2003). We next performed *bmp4* induction at 8.5 hpf for 15, 10, and 7.5 min (Figure 2A, scheme of experimental procedure) and addressed the expression of *cryaa* by whole‐mount in situ hybridization (WMISH). *Cryaa* is a gene coding for a crystallin protein, important for lens development and function. *Cryaa* expression was regularly found in lenses of control embryos (Figure 2B,C). *Cryaa* expression could also be detected in *bmp4*‐induced embryos (Figure 2D–J, metadata can be found in the Supporting Information Table). After induction of *bmp4* for 15 min, *cryaa* expression was found in patches in an anterior domain (Figure 2D,E). This domain corresponds well to the region where a lens would have been localized in a cyclopic embryo; yet, no proper lens was formed, and importantly, the crypt‐oculoid was not transformed into an optic cup. After induction for 10 min, *cryaa* expression varied with respect to the spectrum of observed phenotypes. *Cryaa* was found expressed in lenses, if these were formed, but also in various ectopic regions, for example, in anterior regions of the head domain (Figure 2F,G). After induction for 7.5 min, *cryaa* expression was found in lenses (Figure 2H,I).

### 2.3 The Basal Constriction of Retinal Progenitors Is Affected by *bmp4*‐Induction

**FIGURE 3:**
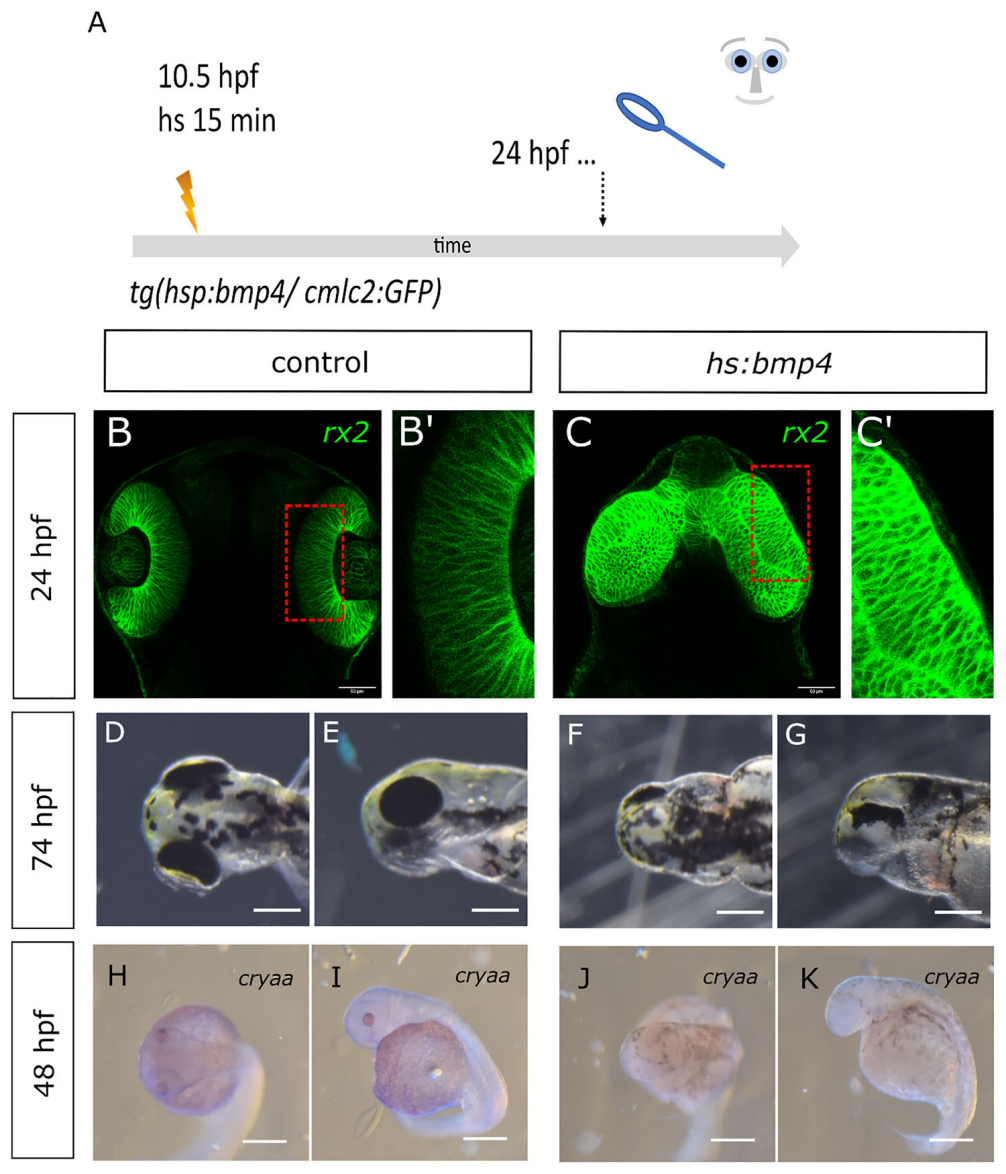
Delayed *bmp4* induction results in “flat eyes.” (A) Summary of experimental procedure: embryos were heat‐shocked at 10,5 hpf (delayed) for 15 min and analyzed 24 hpf or at later stages. (B–Cʹ) Confocal images (dorsal view), with green indicating *rx2*‐positive cells at 24 hpf. (C, Cʹ) Control embryo. (B, Bʹ) *bmp4*‐induced embryo. Please note that the delayed induction resulted in out‐pocketing of optic vesicles, which, however, were not transformed into optic cups. Scale bars indicate 50 µm. (D–G) Gross analysis of embryos at 74 hpf. (D, E) Control embryo. (D) Dorsal view, head left. (E) Lateral view, head left. (F, G) *bmp4*‐indced embryo. (F) Dorsal view, head left. (G) Lateral view, head left. Please note that the induced embryos show a phenotype reminiscent of a flat‐eye “ojoplano” phenotype (Martinez‐Morales et al. 2009). Scale bars indicate 250 µm. (H–K) WMISH for cryaa at 48 hpf. (H, I) Control embryo. (H) Dorsal view, head left. (I) Lateral view, head left. (J, K) *bmp4*‐induced embryo. (J) Dorsal view, head left. (K) Lateral view, head left. Please note that the embryo shows no eyes and no visible *cryaa*‐positive tissue. Scale bars indicate 250 µm.

We next aimed to further address why the crypt‐oculoid does not form an optic cup in *bmp4*‐induced embryos (Figure 2A,D,E). During normal eye morphogenesis, individual retinal progenitors undergo a change of shape, turning them from a columnar shape into a wedge shape. This process is driven by basal constriction (Martinez‐Morales et al. 2009). We used the transgenic line *Tg(Ola.Rx2:EGFP‐CAAX,myl7:EGFP)* to visualize the retinal progenitors with confocal microscopy. We crossed this line with the *bmp4*‐inducible line *Tg(hsp70l:bmp4,myl7:EGFP)*. To be able to address whether the change of shape of individual retinal progenitors is potentially affected by *bmp4* induction, we slightly delayed the *bmp4* induction. We induced *bmp4* via heat shock at 10.5 hpf for 15 min (Figure 3A, scheme of experimental design). At this stage, eye‐field splitting and optic vesicle out‐pocketing have already started. Thus, it was possible to observe the effect of *bmp4* on optic cup formation independently. At 24 hpf, regular optic cups could be observed in control embryos (Figure 3B,Bʹ, 33 embryos). The basal surface, directed toward the lens, showed a typical bending (Figure 3Bʹ). Optic vesicle‐like structures were found after the delayed *bmp4*‐induction (Figure 3C,Cʹ, 29 embryos). The surface of these vesicles, however, remained flat (Figure 3Cʹ), indicating that the shape change of the retinal progenitors was hampered. Furthermore, the vesicle‐like structures were not separated but fused in the midline (Figure 3C). At 74 hpf, the phenotype morphologically resembled the opo mutant phenotype in which the hampered basal constriction was first described (Martinez‐Morales et al. 2009) (Figure 3F,G, 67 embryos, please see D and E as control, 39 embryos). Besides, five embryos with severe degeneration were found (four dead at 4 dpf, genotyping failed; one showed late onset of *myl7:GFP* expression). We next addressed the expression of *cryaa* in the delayed induction paradigm. Unexpectedly, we did not detect *cryaa* expression after *bmp4* was induced at 10.5 hpf for 15 min (Figure 3J,K, three embryos, please see H and I as control).

### 2.4 *Vsx2*, *ofcc1*, and *lhx2*b Expressed in Crypt‐Oculoids

**FIGURE 4:**
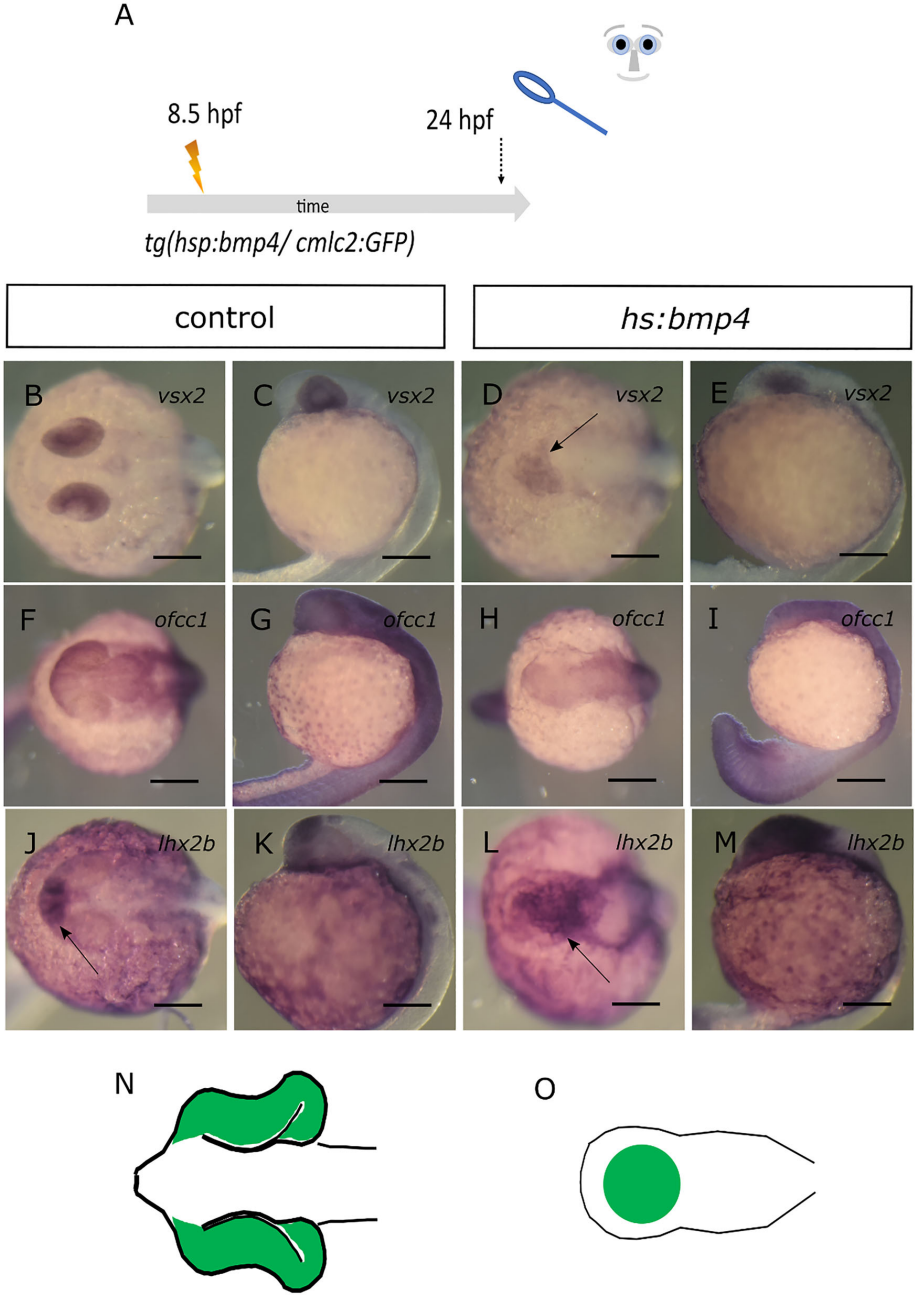
*Vsx2*, *ofcc1*, and *lhx2b* are expressed in crypt‐oculoids. (A) Summary of experimental procedure: embryos were heat‐shocked at 8.5 hpf for 15 min and analyzed 24 hpf. (B–E) WMISH for *vsx2*. (B, C) Control embryos. (B) Dorsal view, head left. (C) Lateral view, head left; *vsx2* expression in optic cups. (D, E) *bmp4*‐induced embryos. (D) Dorsal view, head left. (E) Lateral view, head left; *vsx2* expression in the head and crypt‐oculoid (arrow). (F–I) WMISH for *ofcc1*. (F, G) Control embryos. (F) Dorsal view, head left. (G) Lateral view, head left; *ofcc1* expression in optic cups and other domains. (H, I) *bmp4*‐induced embryos. (D) Dorsal view, head left. (E) Lateral view, head left; *ofcc1* expression in the head and crypt‐oculoid. (J–M) WMISH for *lhx2b*. (J, K) Control embryos. (J) Dorsal view, head left. (K) Lateral view, head left; *lhx2b* expression in telencephalon, diencephalon, and optic cups (arrow). (L, M) *bmp4*‐induced embryos. (L) Dorsal view, head left. (M) Lateral view, head left; *lhx2b* expression in the head and crypt‐oculoid (arrow). (N, O) Scheme of phenotype of control (N) and *bmp4*‐induced embryo (O); “eye tissue” is marked in green. Scale bars indicate 200 µm.

The shape change of the progenitors during optic cup morphogenesis is controlled by *opo/ofcc1* (Bogdanović et al. 2012; Martinez‐Morales et al. 2009). V*sx2* was found as an upstream regulator of *ofcc1* (Gago‐Rodrigues et al. 2015). For the next steps of our analyses, we went back to the original, early treatment paradigm of *bmp4* induction at 8.5 hpf for 15 min (Figure 4A, scheme, all embryos expressing *myl7:eGFP* showed anophthalmia phenotype). We addressed whether the expression of *vsx2* and *ofcc1* is affected by the induction of *bmp4*. To facilitate the interpretation of expression pattern changes, we included a scheme of the morphological changes of the embryo (Figure 4O, please see N as control). In control embryos, *vsx2* expression was detected in optic cups (Figure 4B,C, 10 control embryos). In *bmp4*‐induced embryos, *vsx2* expression was found in the head region and the crypt‐oculoid (Figure 4D,E, 10 induced embryos). In control embryos, *ofcc1* can be found expressed in the optic cups among many other domains (Figure 4F,G, five control embryos). *Ofcc1* expression was found in the region of the crypt‐oculoid after *bmp4* induction (Figure 4H,I, four induced embryos). The transcription factor *lhx2b* was also found essential for the transformation of the optic vesicle into the optic cup (Porter et al. 1997). We thus also addressed *lhx2b* expression. In control embryos, *lhx2b* expression could be detected in the telencephalon, diencephalon, and ventral optic cup (Figures 4J,K and S4, five control embryos). After induction of *bmp4*, the expression of *lhx2b* could be detected in the head and the crypt‐oculoid (Figure 4L,M, five induced embryos). Together, we could not detect a clear suppression of expression of either *ofcc1*, *vsx2*, or *lhx2b*. These findings suggest that the process of basal constriction of the retinal progenitors is more likely affected by other means than by the level of expression of these genes.

## 3 Summary and Discussion

**FIGURE 5:**
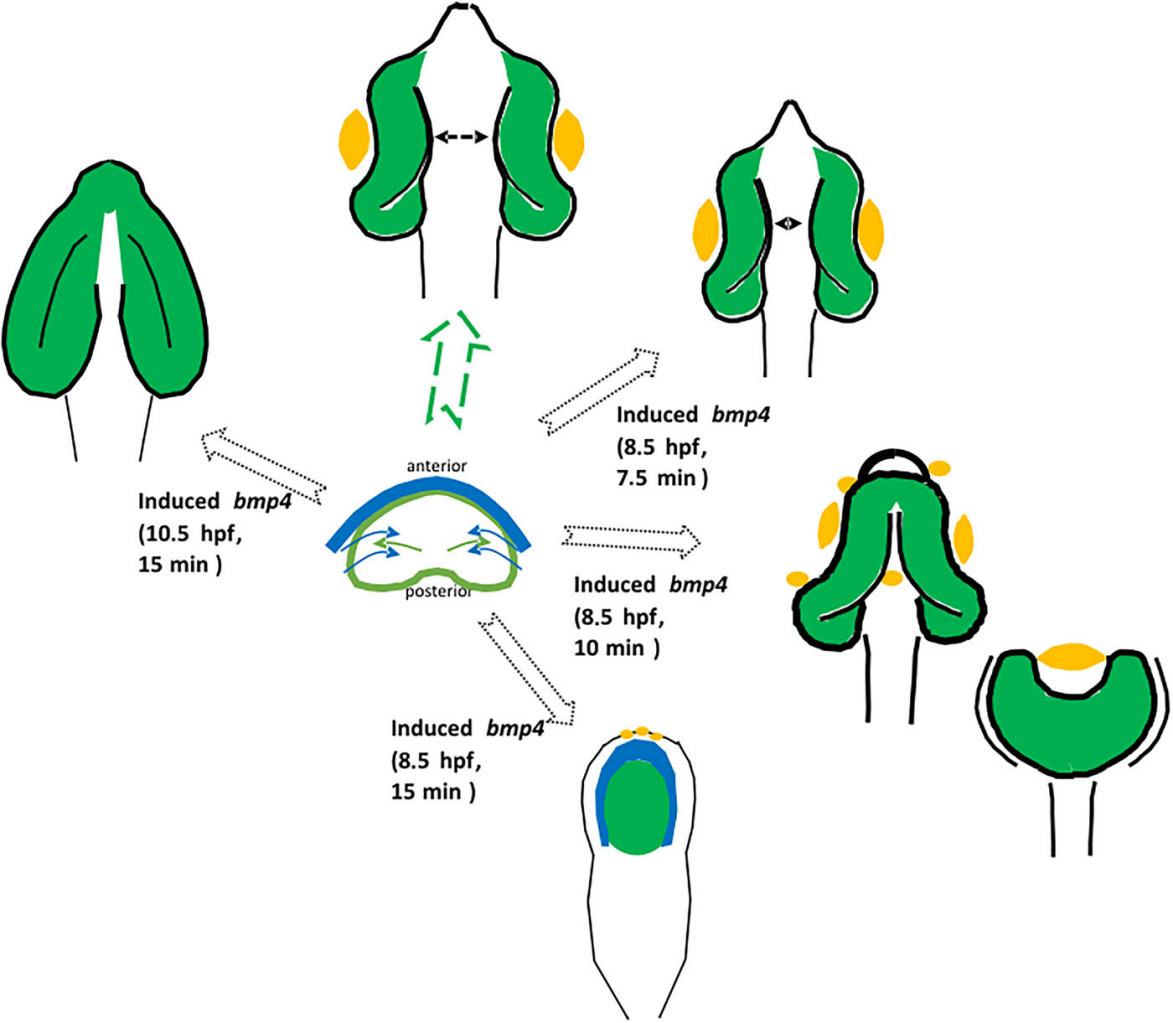
Schematic summary of findings. The eye field (green) within the ANP (center) is split and develops (green arrow) into bilateral optic cups (top), including lenses/cryaa expression domains (yellow) (arrow marks distance between optic cups). *Bmp4* induction at 8.5 hpf for different durations results in graded phenotypes (7.5 min top right: ocular hypotelorism, 10 min middle right: synophthalmia/cyclopia, 15 min bottom right: crypt‐oculoid). Delayed induction of *bmp4* (top left) hampers optic cup bending and results in flat eyes. Blue: forebrain.

In this study, we used an inducible HPE model to address ophthalmologic phenotypes. The HPE model was established recently and is based on a heat‐shock‐driven expression of *bmp4* in zebrafish. Previous work has demonstrated that induced expression results in enhanced BMP signaling (Bulk et al. 2023; Eckert et al. 2020). Induction at 8.5 hpf, a developmental stage during gastrulation (Kimmel et al. 1995), for the duration of 15 min hampered the division of both the telencephalic domain and the eye field of the early forebrain (prosencephalon) and thus resulted in HPE. *Bmp4* induction likely oversaturated endogenous BMP antagonists within and around the ANP (e.g., *nog2*, *chrd*, *fsta*, and *grem2b*). The ophthalmologic finding that resulted from the hampered division was anophthalmia. Importantly, retinal precursors were found inside a dysmorphic forebrain, termed crypt‐oculoid (Bulk et al. 2023) (Figure 5, schematic summary).

HPE is the most frequent developmental forebrain pathology in humans (Malta et al. 2023; Matsunaga and Shiota 1977; Pineda‐Alvarez et al. 2011), with an incidence of approximately one in 10,000 at birth and 0.4% at conception (Matsunaga and Shiota 1977). It has been graded in groups of severe forms (alobar HPE), intermediate forms (semilobar HPE), and less severe forms (lobar HPE) (Gomez et al. 2024). The ophthalmologic phenotypes that accompany HPE can be found as a spectrum, including coloboma, ocular hypotelorism, synophthalmia, cyclopia (Pineda‐Alvarez et al. 2011), and anophthalmia (Fallet‐Bianco 2018). Phenotypes of this spectrum were observed in the present analysis (Figure 5, schematic summary).

Notably, the diagnosis “anophthalmia” has to be parsed. It is used in the context of the MAC spectrum (microphthalmia, anophthalmia, and coloboma) (Ohuchi et al. 2019). In this context, anophthalmia results from pathologies during the development of the optic vesicles, similar to the evolutionary loss of eyes in cavefish (Ohuchi et al. 2019). In these cases, the eye field was already separated. As beforementioned, anophthalmia, however, can also be part of HPE (Fallet‐Bianco 2018). Here, anophthalmia means that the eye field does not split and that no optic vesicles develop in the first place. The form of HPE that underlies this eye phenotype is considered very severe. The prosencephalon is largely missing, termed pseudo‐aprosencephaly (Fallet‐Bianco 2018; Schmitt and Born 1988). Anophthalmia is thus described as “most severe” in the context of HPE (Fallet‐Bianco 2018). Interestingly, a double knockout of two BMP antagonists in mice (chordin and noggin) variably affected forebrain development, and cases of both cyclopia and aprosencephaly were described (Bachiller et al. 2000).

Here, we aimed to investigate whether, by attenuation of the induction regime, other ophthalmologic phenotypes could be found. It is to be stated that an inducible expression system, based on the heat‐shock promoter–driven expression, does not allow the control of exact expression levels. The aim of this study was to simply reduce the level of expression. To this end, the heat shock length was reduced—10 and 7.5 min were used instead of 15 min. Subsequently, a spectrum of phenotypes—cyclopia, synophthalmia (10 min), and ocular hypotelorism (7.5 min)—was observed. Schematic representations of these are summed up in Figure 5. These findings are consistent with the graded eye phenotypes shown by previous work (Fallet‐Bianco 2018; Pineda‐Alvarez et al. 2011).

Future analyses are needed to resolve which exact levels of *bmp4* result in which of these ophthalmologic phenotypes. This must be investigated in the context of the different BMP antagonists, expressed around and within the ANP (Bulk et al. 2023), and different BMP signaling pathways (canonical and noncanonical). Furthermore, the mechanism by which BMP signaling affects morphogenesis is still elusive, and the molecular changes that result from the various *bmp* expression levels must be addressed.

A known motor that drives morphogenesis of the optic cup is *ojoplano* (*opo*) (Martinez‐Morales et al. 2009). *Opo* is needed to reshape retinal precursors from a column into a wedge. To address whether this process is affected by induced levels of *bmp4*, we needed to delay the induction (10.5 hpf) to give the eye field time to out‐pocket. This way, we were able to show that the delayed induction impeded the reshaping. The phenotype that resulted from this regime (flat eye) resembled the *opo* mutant and showed only a small dorsal domain of pigmentation (Martinez‐Morales et al. 2009). The development of the retinal pigment epithelium is a fascinating process. In chick, at the optic vesicle stage, BMPs emanating from the surface ectoderm are important for retinal pigment epithelium specification (Müller et al. 2007). Later, implantation of BMP‐soaked beads into the optic cup resulted in trans‐differentiation/conversion of neuroretinal domains into retinal pigment epithelium (Steinfeld et al. 2017). In mice, Bmp4 emanating from the optic vesicle/cup was found to be important for lens induction, while it suppresses corneal development (Huang et al. 2015). Huang and colleagues also found reduced proliferation in the presumptive neuroretinal domain of conditional knockouts for Bmp4 in optic vesicle tissue. These findings, however, were interpreted as a fate switch to retinal pigment epithelium, in which the proliferation at that developmental stage was also reduced in controls, supported by gene expression data. Thus, a potential different role of BMPs in the specification of retinal, retinal pigment epithelial, and neuroretinal tissues in mouse and chick was deduced (Huang et al. 2015). It has to be noted, though, that neither model system allowed addressing in vivo imaging of optic‐vesicle‐to‐optic‐cup transformation. In zebrafish, however, this was possible and allowed the identification of a highly dynamic process (Heermann et al. 2015; Kwan et al. 2012; Picker et al. 2009; Sidhaye and Norden 2017). This dynamic “bilateral neuroretinal flow” or “rim movement” during optic‐vesicle‐to‐optic‐cup transformation is also important to consider when interpreting data of mutants and overall data of gene expression over time. Gene expression or signaling domains may be localized in ectopic regions due to hampered cellular motility and not necessarily exclusively due to misspecification (Eckert et al. 2019). This is important to consider for analysis of data during morphogenesis and less important at later stages, for example, at optic cup stages as mentioned above (Steinfeld et al. 2017). Currently, we can only speculate to what extent changes in morphogenesis and specification contribute to the phenotype we observed after delayed *bmp4* induction (Figure 3). Our previous data in the context of BMP induction and eye morphogenesis indicate, however, that morphogenesis/cellular migration at different consecutive stages of eye morphogenesis is protected by BMP antagonists that are expressed in specific domains (Eckert et al. 2019; Heermann et al. 2015; Knickmeyer et al. 2018). In Heermann et al. (2015), we found that induced BMP induction arrested a highly dynamic bilateral “neuroretinal flow,” which in control embryos brings future neuroretinal cells to the inside (lens‐facing) layer during optic‐vesicle‐to‐optic‐cup transformation. Only later *ojoplano* function was linked to the “neuroretinal flow,” also called “rim movement” (Sidhaye and Norden 2017). It is thus reasonable to assume that the flat eye we observed, which resulted from the delayed *bmp4* induction and is reminiscent of the *opo* mutant, is the effect of hampered morphogenesis rather than a fate conversion, at least to a large extent. It is elusive how exactly this *opo*‐mediated reshaping is influenced by *bmp4* induction and whether this kind of reshaping is even a driver of eye‐field splitting or optic vesicle out‐pocketing. Neither *opo* nor the upstream regulator *vsx2* was found absent after early induction (8.5 hpf). In addition, *lhx2b*, another important factor for optic cup morphogenesis (Porter et al. 1997), was expressed after *bmp4* induction. This suggests that these factors are at least not involved via transcriptional regulation. Even though these findings do not add to the understanding of why crypt‐oculoids are formed, the data do add to the understanding of the “bilateral neuroretinal flow” (Heermann et al. 2015) or “rim involution” (Kwan et al. 2012; Sidhaye and Norden 2017) found during optic‐vesicle‐to‐optic‐cup transformation. Previous data demonstrated that this process depends on *opo* (Sidhaye and Norden 2017) and is stopped by induced levels of *bmp4* (Heermann et al. 2015).

Previous work has shown that the interplay of pre‐lens ectoderm and neuroectoderm is essential for optic cup development (Hyer et al. 2003). Following *bmp4* induction (8.5 hpf for 15 min), we found lens tissue in a central anterior expression domain (Figure 5, schematic summary, marked in yellow). This might be interpreted as part of an anlage of a cyclopic eye. Nevertheless, such a cyclopic eye was not formed in consecutive stages of development in these embryos. This suggests that the interaction between lens tissue and neuroectoderm failed here. To our surprise, lens tissue could not be identified in the delayed induction regime (10.5 hpf) (Figure 5, schematic summary). In this delayed regime, it seems possible that the fact that lens tissue is absent itself is functionally relevant for the flat eye phenotype (*opo*‐like).

We can speculate how the induction of *bmp4* results in these phenotypes and which other signals may be involved. The absence of lens tissue, as well as the appearance of ectopic lens tissue, is of special interest. Several signaling pathways are important for lens induction, for example, Wnt, FGF, and BMP. The role of Wnt signaling is intriguing, as it is important to restrict lens induction to the correct area. Conditional loss of beta‐catenin, crucial for canonical Wnt signaling, in periocular ectoderm results in ectopic “lentoid bodies” that are beta‐crystallin positive (Smith et al. 2005). Later, on the other hand, beta‐catenin is important for lens morphogenesis (Smith et al. 2005). Thus, it is conceivable that *bmp4* induction in our experiments resulted in the inhibition of canonical Wnt signaling in the periocular ectoderm. A plethora of Wnt ligands exist that signal not only via canonical but also via diverse noncanonical Wnt signaling pathways (Qin et al. 2024). Wnt5a, for example, was found to be important for lens formation in vitro, likely via noncanonical signaling (Han et al. 2018). Besides Wnt, FGF signaling is also important during early lens development (Cvekl and Zhang 2017). BMP induction might thus also interfere with FGF signaling. It was shown that Bmp7 is important for early lens formation (Wawersik et al. 1999) and interacts with FGF signaling (Faber et al. 2001; Lang 2004). In our experiments, we induced *bmp4*. It has been shown previously that BMP4 and BMP7 can act as heterodimers (Aono et al. 1995), but other, even contrary, interactions between BMP ligands and their signaling are also possible. In zebrafish, induction of *bmp4* can transcriptionally downregulate specific BMP receptors and ligands, for example, *bmp7b* and *bmpr1ba* (Knickmeyer et al. 2021). Conversely, in mice, conditional loss of Bmp4 in the optic vesicle/cup results in transcriptional upregulation of Bmp7 (Huang et al. 2015). Besides these speculative direct interactions between induced levels of *bmp4* and lens development, it is, however, also conceivable that the effects we observed are rather indirect, potentially mediated by altered cell populations.

Overall, our data suggest that the two processes, eye‐field splitting and optic cup development, are influenced by common elements (BMP signaling/ antagonism) and, however, also by other factors that are specific (lens).

## 4 Materials and Methods

### 4.1 Zebrafish Care

Adult zebrafish were kept in accordance with the local animal welfare law and with the permit from the “Regierungspräsidium Freiburg”: 35–9185.64/1.1. Fish were housed in a recirculating system at 28°C in a 12‐h light/12‐h dark cycle. The following transgenic lines were used: tg(hsp70l:bmp4, myl7:eGFP) (Knickmeyer et al. 2018) (RRID:ZFIN_ZDB‐ALT‐180816‐2) and tg(Ola.Rx2:EGFP‐CAAX, myl7:EGFP) (Heermann et al. 2015) (RRID:ZFIN_ZDB‐ALT‐150610‐2). Zebrafish embryos were grown in Petri dishes containing zebrafish medium (0.3 g/L sea salt in deionized water). Melanin‐based pigmentation was inhibited if needed for downstream applications. To this end, embryos were incubated in 0.2 mM phenylthiourea.

### 4.2 Heat Shock Procedures

The application of a heat shock was used for heat‐shock‐inducible transgenes. Embryos at different developmental stages were incubated in 1.5‐mL reaction tubes at 37°C (heating block, Eppendorf Thermomixer). Onset and duration of the heat shock are indicated in the respective experimental setup.

### 4.3 In Situ Hybridization

WMISHs were performed as described previously (Bulk et al. 2023; Quiring et al. 2004). We digested fixed embryos with 10 µg/mL Proteinase K following hybridization with digoxigenin‐labeled RNA probes in PTW using an Intavis InsituPro VSi. For staining, we incubated the samples with anti‐digoxigenin antibody (1:2000 dilution) and developed the color using nitroblue tetrazolium chloride/5‐bromo‐4‐chloro‐3‐indolylphosphate (NBT/BCIP).

RNA probes were amplified from cDNA with Taq Polymerase (New England Biolabs) and then cloned using the pGEM‐T Easy Vector System I. In vitro transcription was performed using SP6 or T7 RNA polymerase.

The following probes were used: *vsx2* (Eckert et al. 2020), probe length 750 bp; *ofcc1* F: GATGCTGCCAAGCTCTACTGG, R: TTCATCCTCTCGTGTTGCTCAT, probe length 542 bp; and *lhx2b* F: ATGCCTTCAATCAGCGGG, R: TCAGAAGAGGCTGGTTAAGG, probe length 1065 bp. Probes for *cryaa* were designed cloning‐free (Hua et al. 2018). Sequences are as follows: *cryaa* F: CCAACACCCTTGGTTCAGAC, *cryaa* R: GTAACAGGGATGGTGCGATCT, probe length 471 bp.

### 4.4 Image Processing

Images were recorded via stereomicroscopy (Nikon SMZ 18) and confocal microscopy (Leica TCS SP8). Images were subsequently edited for presentation using ImageJ (Fiji) (Schindelin et al. 2012) and Inkscape (Inkscape Project 2020; https://inkscape.org).

## Author Contributions

Johannes Bulk performed experiments (heat shock induction, image acquisition, in situ hybridization, imaging), data visualization, and manuscript review. Valentyn Kyrychenko performed experiments (in situ hybridization, image acquisition), data visualization, and manuscript review. Stephan Heermann performed conceptualization and visualization of data and schemes, provided resources, prepared the original draft and revisions, and carried out supervision and project administration.

## Conflicts of Interest

The authors declare no conflicts of interest

## Supporting information




**Supplementary Figure**: cne70113‐sup‐0001‐figureS1.tif

**Supplementary Figure**: cne70113‐sup‐0002‐figureS4.png

**Supplementary Table**: cne70113‐sup‐0003‐tableS1.docx

**Supplementary Material**: cne70113‐sup‐0004‐SuppMat.docx

## Data Availability

Data sharing not applicable to this article as no datasets were generated or analyzed during the current study.
