# Supplementary figures and images for: Ophthalmologic Findings in an Induced Model of Holoprosencephaly in Zebrafish
Source: J Comp Neurol. 2025 Nov 9;533(11):e70113. doi: 10.1002/cne.70113 (PMC12597868; doi:10.1002/cne.70113)

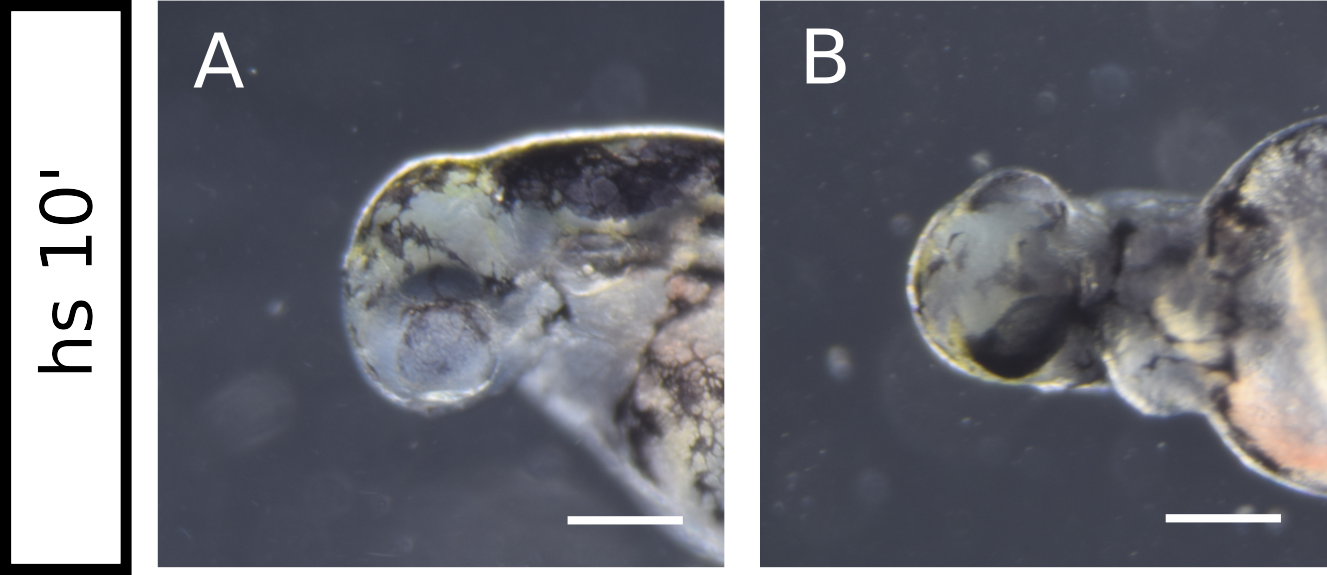

Supplement: Supplementary file 1 — Supplementary Figure: cne70113‐sup‐0001‐figureS1.tif [file CNE-533-e70113-s004.tif]

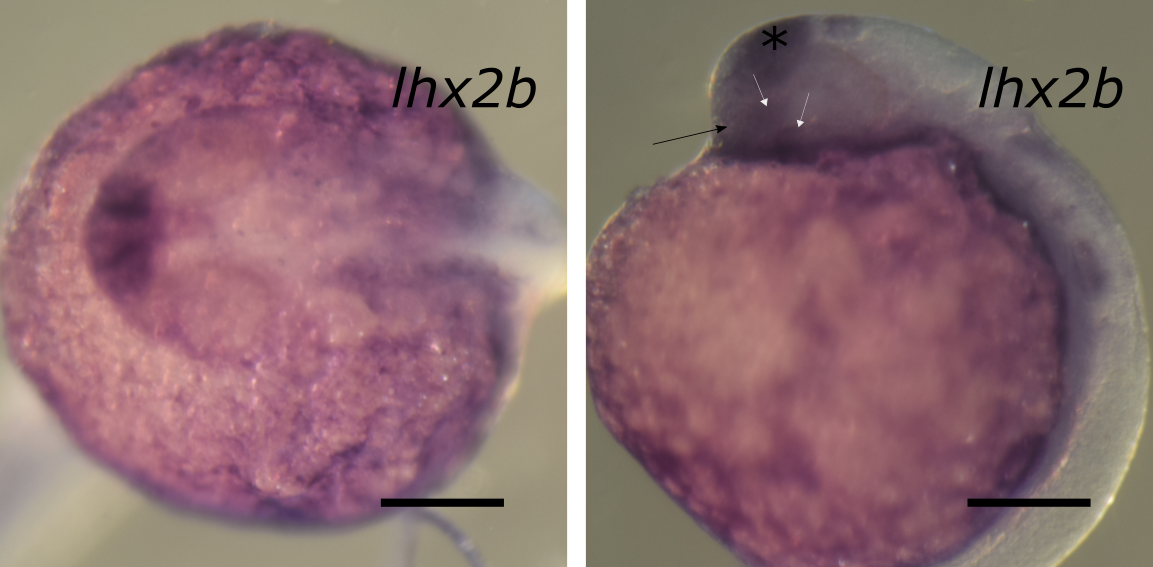

Supplement: Supplementary file 2 — Supplementary Figure: cne70113‐sup‐0002‐figureS4.png [file CNE-533-e70113-s002.png]
