# Supplementary material for: Ophthalmologic Findings in an Induced Model of Holoprosencephaly in Zebrafish
Source: J Comp Neurol. 2025 Nov 9;533(11):e70113. doi: 10.1002/cne.70113 (PMC12597868; doi:10.1002/cne.70113)
Supplement: Supplementary file 3 — Supplementary Table: cne70113‐sup‐0003‐tableS1.docx [file CNE-533-e70113-s003.docx]

| Figure | Intervention | Time point of heat shock | Duration of heat shock | Transgene (hsp70:bmp4/ cmlc2:GFP) | Phenotype as described | No phenotype (wild-type pattern) |
| --- | --- | --- | --- | --- | --- | --- |
| 1 | Observation 3 dpf | 8.5 hpf | 10 min | - | - | 198 |
|  |  |  |  | + | 186 | - |
|  |  |  | 7.5 min | - | 1 | 36 |
|  |  |  |  | + | 40 | - |
|  | ISH cryaa 48 hpf |  | 15 min | - | - | 2 |
|  |  |  |  | + | 10 | - |
|  |  |  | 10 min | + | 9 | - |
|  |  |  | 7.5 min | + | 8 | - |
| 2 | Evaluation with trans-gene rx2:GFP | 10.5 hpf | 15 min | - | - | 33 |
|  |  |  |  | + | 29 | - |
|  | Observation at 74 hpf |  |  | - | 5 | 39 |
|  |  |  |  | + | 67 | - |
|  | ISH cryaa 48 hpf |  |  | + | 3 | - |
| 3 | ISH ofcc1 24 hpf | 8.5 hpf | 15 min | - | - | 5 |
|  |  |  |  | + | 4 | - |
|  | ISH vsx2 24 hpf |  |  | - | - | 10 |
|  |  |  |  | + | 10 | - |
|  | ISH lhx2b 24 hpf |  |  | - | - | 5 |
|  |  |  |  | + | 5 | - |

Supplemental Table

Metadata for experimental setups including heat shock lengths, onsets and outcome.
