# Supplementary material for: Ophthalmologic Findings in an Induced Model of Holoprosencephaly in Zebrafish
Source: J Comp Neurol. 2025 Nov 9;533(11):e70113. doi: 10.1002/cne.70113 (PMC12597868; doi:10.1002/cne.70113)
Supplement: Supplementary file 4 — Supplementary Material: cne70113‐sup‐0004‐SuppMat.docx [file CNE-533-e70113-s001.docx]

**Legends to Figure Supplements**

Figure 1 supplement. Spectrum of ocular phenotypes. A and B: *bmp4*-induction for 10 min. Optic cups are fused in the middle resembling a strong case of synophthalmia. A: lateral view, head left, B: ventral view, head left. Scale bars indicate 250 μm.

​​

Figure 4 supplement. Magnification of Figure 4 J and K showing the expression of lhx2b in a control embryo. Expression can be seen in the telencephalic region (asterisk), a hypothalamic domain (black arrow) and in the ventral optic cup (fissure margins, white arrows). Scalebars indicate 200 µm.
